# Monitoring the Surface Energy Change of Nanoparticles in Functionalization Reactions with the NanoTraPPED Method

**DOI:** 10.3390/nano13071246

**Published:** 2023-03-31

**Authors:** Andrei Honciuc, Oana-Iuliana Negru

**Affiliations:** Petru Poni Institute of Macromolecular Chemistry, Aleea Gr. Ghica Voda 41A, 700487 Iasi, Romania

**Keywords:** surface energy, contact angle, nanoparticles, interfaces, Pickering emulsions, interfacial energy, self-assembly of nanoparticles

## Abstract

Performing chemical functionalization on the surface of nanoparticles underlies their use in applications. Probing that a physicochemical transformation has indeed occurred on a nanoparticles’ surface is rather difficult. For this reason, we propose that a macroscopic parameter, namely the surface energy γ, can monitor the physicochemical transformations taking place at the surface of nanoparticles. Determining the surface energy of macroscopic surfaces is trivial, but it is very challenging for nanoparticles. In this work we demonstrate that the Nanoparticles Trapped on Polymerized Pickering Emulsion Droplet (NanoTraPPED) method can be successfully deployed to monitor the evolution of surface energies γ, with its γ^p^ polar and γ^d^ dispersive components of the silica nanoparticles at each stage of two surface reactions: (i) amination by siloxane chemistry, coupling reaction of a 2,4-dihydroxy benzaldehyde and formation of a Schiff base ligand, followed by coordination of metal ions and (ii) epoxide ring opening and formation of azide. The change in surface energy and its components are discussed and analyzed for each step of the two reactions. It is observed that large variations in surface energy are observed with the complexity of the molecular structure attaching to nanoparticle surface, while functional group replacement leads to only small changes in the surface energies.

## 1. Introduction

**Scheme 1:**
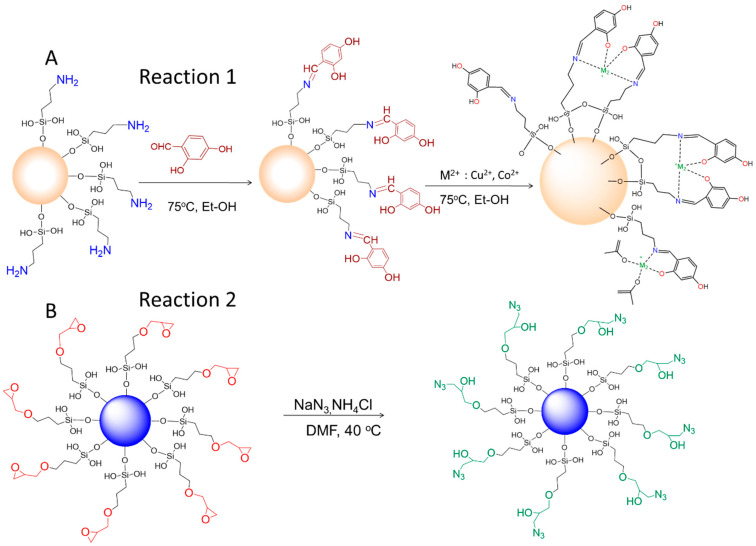
(**A**) Formation of Schiff base interfacial ligand, NP-L, by condensation of the 2,4-dyhydroxy benzaldehyde with the amine surface functionalities, NP-NH_2_, and formation of the complex by coordination of a divalent metal ion Cu(II) or Co(II), giving rise to NP-LCu and NP-LCu. (**B**) Functional group replacement reaction of glycidyl with azide by ring opening and substitution reaction.

The functional groups present onto the surface of nanoparticles (NPs) determine the way the NPs interact with the environment, controlling their behavior in bulk powders but also in liquid or gaseous media, especially their chemical reactivity. Performing surface chemical modification on the surface of nanoparticles to make them useful in a variety of applications has become ubiquitous and routine [1]. The chemical transformations taking place at the surface of nanoparticles following chemical treatment, transformation of chemical functional groups via oxidation, coupling, condensation, polymerization reactions, or even physical adsorption and coordination of metal ions, is difficult to monitor but can be done in principle by employing advanced surface specific spectroscopy methods. Although one can enumerate some surface sensitive spectroscopy methods capable of resolving chemical composition and surface functional groups, these are generally difficult to deploy on powders consisting of nanoparticles. However, even though the presence of a certain functional group, of several or combinations thereof on the surface of NPs could be resolved through various spectroscopy methods, this gives little information on the actual physicochemical behavior of the nanoparticle in bulk powder or its preferred way to interact with the environment. In an earlier work, we have alluded at the possibility that a macroscopic parameter, namely the surface energy, which describes the capacity of a surface to interact with its environment through various physicochemical forces [2], can serve as one of the essential parameters to describe the physicochemical state of the NPs, and could help establish a causal relationship with the NPs’ behavior in bulk to help design powders with good flowing ability [3], improve dispersion of pigments in polymer matrices [4], understand powder cohesion [5], flotation of ore and minerals [6], emulsification and creation of Pickering emulsions [7], stability of colloids [2], etc. Even more, based on the governing principle of independent actions, the total surface energy γ, regardless of scale, can be seen as the sum of various components that reflect the capacity of a surface to interact with the environment through different kind of interaction forces, such as dipole–dipole γ^p^, dispersive van der Waals interaction forces γ^d^, hydrogen bonding γ^H−H^, acid γ^A^ and base γ^B^, and so on. This constitutes a further advantage in the holistic characterization of the physicochemical state of NPs as the relative magnitude of the surface energy components provide meaningful information of the preferred type of interaction of a particular surface. For a macroscopic surface, the surface energy and its polar and dispersive components γ, γ^p^ and γ^d^ can be determined from the contact angle of at least two liquid droplets, see the Owens–Wendt–Rabel–Kaelble (OWRK) model [8]. However, in case of nanoparticle surfaces, making droplets smaller than the nanoparticles to determine their contact angle is technically very challenging. Although some methods have been developed to specifically address the measurement of the contact angle of a single NP with a liquid [9,10,11], none could be deployed yet in measurement of the contact angles of NPs with multiple liquids, which is necessary to determine the surface energy and its components. To overcome this, we have implemented a new method called Nanoparticles Trapped on Polymerized Pickering Emulsion Droplet (NanoTraPPED), and demonstrated that this method is capable of measuring the contact angles of NPs at various oil/water interfaces. Additionally, from these values of interfacial energies, the surface energies with the polar and disperse components can be determined [2]. We have shown that water/NP interfacial energies obtained with NanoTraPPED are extremely useful at predicting the dispersibility and stability of various NPs in water, while the surface energies are useful in predicting their emulsification ability and monitoring the physicochemical state of NPs in air. In this work we demonstrate that the NanoTraPPED method can be successfully deployed to monitor the evolution and change of interfacial NP/water energies $\gamma_{NP/water}$, $\gamma_{NP/water}^{p}$ and $\gamma_{NP/water}^{d}$ and surface energies $\gamma_{NP}$, $\gamma_{NP}^{p}$ and $\gamma_{NP}^{d}$ of fresh silica NPs with a hydroxylated surface (NP-OH). They undergo surface physicochemical transformations, amination by siloxane chemistry (NP-NH_2_), the coupling reaction of a 2,4-dyhydroxy benzaldehyde and the formation of a Schiff base ligand (NP-L), followed by the capturing and coordination of Cu(II) (NP-LCu) or Co(II) (NP-LCu) ions, according to the reactions presented in Scheme 1. Interestingly, the total surface energy and its components increases with the increase in the complexity of the molecular structure of the nanoparticle surface.

## 2. Materials and Methods

### 2.1. Materials

Tetraethylorthosilicate (TEOS) 99% and benzoin methyl ether (BME) 97% were purchased from ABCR; GmbH, 3-(trimethoxysilyl) propylamine 98% (APTES), 3, (3-glycidoxypropyl)trimethoxysilane (GLYMO), divinylbenzene (DVB) technical grade 80%, benzyl metacrylate (BM) 96% containing monomethyl ether hydroquinone as inhibitor, tert-butyl acrylate (tBA) 98% containing 10–20 ppm monomethyl ether hydroquinone as inhibitor, methyl methacrylate (MM) 99% stabilized for synthesis with monomethyl ether hydroquinone as inhibitor, 2-(Dimethylamino)ethyl methacrylate (DAEMA) containing 700–1000 ppm monomethyl ether hydroquinone as inhibitor 98%, ethyl methacrylate (EM) 98% stabilized with 200 ppm hydroquinone, 2,4-dyhydroxy benzaldehyde, ammonium chloride, sodium azide, aluminum oxide (active basic) Brockmann I and N,N-Dimethylformamide anhydrous 99.8% were purchased from Sigma-Aldrich; absolute ethanol (EtOH, 99.3%), hydrochloric acid (HCl), Cu(CH_3_COO)_2_·H_2_O and Co(CH_3_COO)_2_·4H_2_O were purchased from Chemical Company; ammonium hydroxide solution (28–30%) for analysis was purchased from EMSURE ACS. Reag. Ph Eur. Supelco.

### 2.2. Synthesis of Silica Nanoparticles

The preparation procedure for silica nanoparticles (NP) and silica nanoparticles functionalized with amine by reaction with APTES was reported previously [2]; the functionalization with epoxy by reaction with GLYMO was also reported [7].

### 2.3. Synthesis of NP-L, NP-LCu and NP-LCo

NP-Ligand (NP-L) was synthesized using the post-grafting method, as shown in Scheme 1. A total of 1.2 g of NP-NH2 was then refluxed with 50 mg of 2,4-dihydroxybenzaldehyde in ethanol for 24 h at 75 °C. The resulting NP-L dispersion was centrifuged, and the particles were then rinsed three times with ethanol and distilled water. Thereafter, we prepared a dispersion containing 0.5 g NP-L in of 20 mL absolute ethanol over which we added dropwise a solution of 45 mg Cu(CH_3_COO)_2_∙H_2_O dissolved in 10 mL absolute ethanol or 40 mg Co(CH_3_COO)_2_∙4H_2_O dissolved in 10 mL absolute ethanol. The reaction took place at 70 °C for 24 h. After the end of the reaction, the NPs were washed three times with ethanol and three times with distilled water and stored in water for subsequent use in emulsification.

### 2.4. Synthesis of NP-N_3_

NP-N_3_ was obtained following the nucleophilic substitution reaction at the epoxy ring in the presence of NaN_3_ and NH_4_Cl. In a typical procedure, we started with a suspension of 1 g NP-Gly in approx. 40 mL DMF to which we added 32.5 mg (5 mmol) of NaN_3_ and 26.8 mg (5 mmol) of NH_4_Cl. The reaction mixture was allowed to react at 40 °C for 24 h. After the end of the reaction, the nanoparticles were washed three times with ethanol and three times with water and stored in water for subsequent use in emulsification.

### 2.5. Pickering Emulsion Preparation and Polymerization 

Water immiscible vinyl bearing monomers EM, BM, tBA, DAEMA and MM, having different polarities, whose chemical structures are given in Figure S3 in the Supplementary Material, and a crosslinking monomer DVB, were used for the Pickering emulsion preparation and polymerization. The Pickering emulsion was produced by first adding 20 mg of BME radical initiator to a 20 mL glass scintillator vial, followed by 1 mL of monomer and 0.1 mL of DVB crosslinker, followed by 5 min of waiting for the mixture to become homogeneous. Next, 5 mg of colloidal particles and 12 mL of water were added. The glass scintillator vial was then sonicated with a Branson 450 Sonifier equipped with a 7 mm diameter horn for 15 s at 30% amplitude. The Pickering emulsions were next exposed for 1 h to a UV lamp (wavelength = 365 nm, with 4 lamps, each with an intensity = 2.2 mW/cm^2^). The synthesis procedures and recipes for each monomer are listed in Table S2. After the polymerization, the product was filtered and thoroughly washed with approximately 15 mL of ethanol to remove the unreacted monomer, and subsequently dried at room temperature.

## 3. Results

The starting silica NPs were synthesized according to a previously reported method [2], which is a modified version of the Stöber process, see Scheme S1 in the Supplementary Material.

### 3.1. Functionalization and Surface Reactions on Silica Nanoparticles

**Figure 1:**
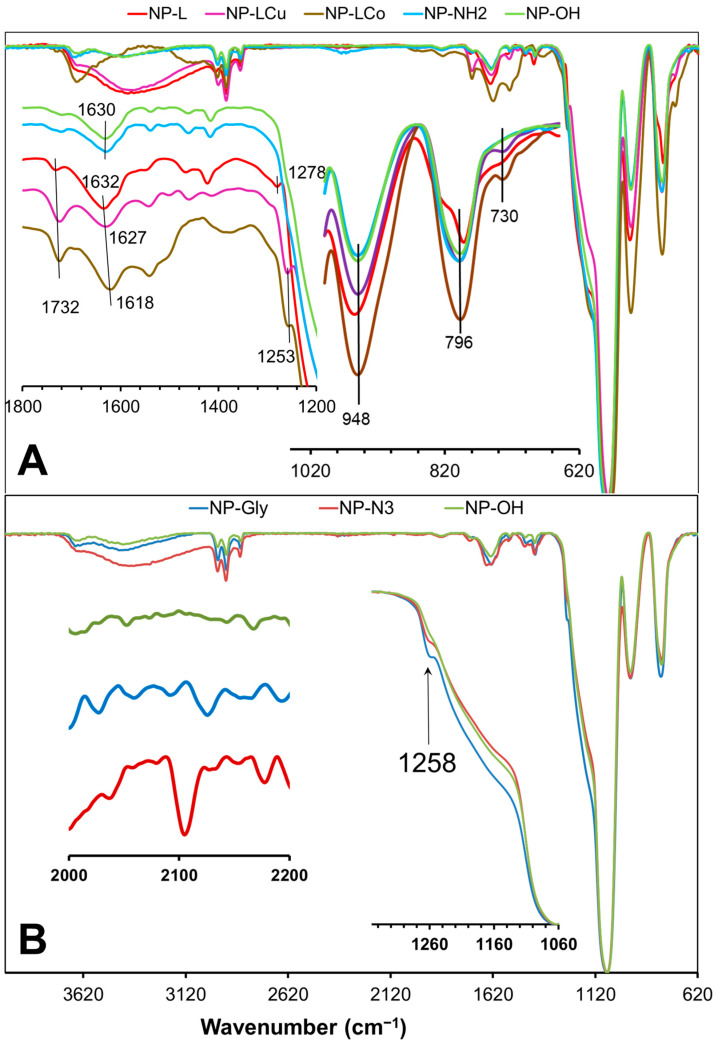
Normalized FTIR spectra of (**A**) Reaction 1: NP-OH (light green), NP-NH_2_ (light blue) and modified NP-L (red), NP-LCu (violet) and NP-LCo (khaki); (**B**) Reaction 2: NP-OH (light green), NP-Gly (blue) and modified NP-N_3_ (crimson). The insets show the offset of the normalized spectra in the regions of interest.

The pristine surface of the silica NPs has mostly hydroxyl surface functionalities, hence the hitherto notation NP-OH. The starting NP-OH have a diameter of ≈500 ± 15 nm, see Figure S1, and were employed in further functionalization such that the subsequent NPs have (i) amine surface functionalities, see Scheme S2, hence the hitherto notation NP-NH2, or (ii) glycidyl functionalities, hence the hitherto notation NP-Gly, see Scheme S2. The presence of the amine functional groups on the NP-NH_2_ can be evidenced both from the ninhydrin reaction, which turns purple in the presence of primary amine, and from the presence of the peaks in the FTIR spectra, see Figure 1. The presence of glycidyl on the surface of the NPs was confirmed with FTIR. We also noted a change in the zeta ζ-potential from −53.7 ± 0.5 mV in NP-OH to lower absolute values −19.4 ± 0.8 mV in NP-NH_2_ at neutral pH, see Table S1. This change in the ζ-potential value can be explained by partial protonation of the amine group even at neutral pH.

Further, the NP-NH_2_ were used in a condensation reaction with 2,4-dyhydroxy benzaldehyde to generate a Schiff base interfacial ligand, NP-L, and subsequent capture and coordination of Cu(II) and Co(II) metal cations (Reaction 1), see Scheme 1A. Alternatively, the NP-Gly were employed in a substitution reaction of the glycidyl group with azide, NP-N_3_, (Reaction 2), see Scheme 1B.

For Reaction 1, the successful functionalization and the formation of the interfacial Schiff base ligand can be confirmed using FTIR, according to the spectra present in Figure 1A. The stretching and bending vibrations of silanol groups (Si-OH) are represented by the bands at 3428 (broad) and 1630 cm^−1^ and are easily identified for the starting NP-OH and NP-NH_2_, Figure 1A. However, with the coupling of the 2,4-dyhydroxy benzaldehyde and formation of a Schiff base ligand, the imine -C=N stretching vibration becomes somewhat visible between 1632 and1618 cm^−1^ Figure 1A. In addition, the formation of the Schiff base ligand is confirmed by the appearance of the several relevant new bands, in NP-L, NP-LCu and NP-LCo at 1461 cm^−1^, which correspond to the aromatic stretching -C=C-. It is also confirmed by the one at 730 cm^−1^, which is due to the deformation vibrations of C-H from the trisubstituted benzene nucleus, as well as the phenolic C-OH stretching vibrations at 1278 cm^−1^. It is interesting to note that the participation of the -OH groups in the coordination of the metal ions is signaled by the shift in the -OH to the vibration of the phenolic group, υ (C-OH), from 1278 cm^−1^ in NP-L, to the lower value of 1253 cm^−1^ in NP-LCu and NP-LCo. The same shift is seen in the stretching frequency of the azomethine υ(C=N) group in the NP-L to lower wavenumbers from 1632 cm^−1^ in NP-L to 1618 cm^−1^ in NP-LCu and NP-LCo complexes due to the coordination of the nitrogen atom of the azomethine group to the metal ion. Peaks in the region of 2900–3200 cm^−1^, corresponding to C-H stretching on NP-OH are mainly due to the incomplete hydrolysis of the precursors, or the capture of the organic hydrolysis fragments into the structure of the NPs; these are always present and disappear only after calcination. For this reason, no spectral interpretation was performed in the region.

Furthermore, the formation of the interfacial Schiff base ligand, is also supported by the change in the zeta potential value, see Table S1, which decreases from −19.4 ± 0.8 mV in NP-NH_2_ to −28.6 ± 0.6 mV in NP-L at normal pH. This evidences even further that the presence of primary amines has been reduced by their participation in the condensation reaction with the 2,4-dyhydroxy benzaldehyde. This is also confirmed by the surface zeta potential change, from −19.4 ± 0.8 mV of the former to a lower surface potential value of −28.6 ± 0.6 mV in the case of the latter. Surface zeta potential decreases to a lower value due to the disappearance of the -NH_2_ and -NH_3_^+^ functionality after the condensation reaction with the aldehyde. Furthermore, the coordination of the Co(II) and Cu(II) ions can be evidenced by a decrease in the zeta potential value from −28.6 ± 0.6 mV in NP-L to −39.6 ± 0.3 mV in NP-LCu and −40.5 ± 0.2 mV in NP-LCo. This can be interpreted as either the coordination of the metal ions that have further diminished the primary amine functional groups on the surface of the NP-L, or that the imine functionalities are also diminished by the coordination of the metal ions. It is worth noting that mere physical adsorption of the divalent metal ions does not produce a dramatic change towards titration of surface charge. This is the case for the trivalent ions [1], as judged from the negative zeta potential values of NPSiCu and NPSiCo, which were NPs left in the presence of a 0.16 × 10^−3^ M solution of Co(II) and 0.25 × 10^−3^ M solution Cu(II) salts and then washed, see Table S1.

For Reaction 2, the successful functionalization and the formation of the NP-Gly and NP-N3 can be confirmed from the analysis of the FTIR spectra, Figure 1B. For example, the shoulder band at 1258 cm^−1^ appearing for the NP-Gly could be identified as the ring vibrations of epoxy, confirming that the surface modification was successful. The epoxides exhibit distinctive IR absorption bands related to ring breathing and asymmetrical ring stretching between 950 and 810 cm^−1^ and 1260 to 1240 cm^−1^. This band at 1258 cm^−1^ is non-existent in NP-OH but can be seen in NP-N3 with a much weaker intensity, suggesting that majority of them had undergone ring opening. The nucleophilic reaction of the epoxy ring with sodium azide is confirmed by the appearance of a small signal in the region of 2200–2050 cm^−1^, which is the characteristic stretching vibration of ν(–N-N≡N) in azide. In addition, changes are observed in the region of 1650–1300 cm^−1^, such as the appearance of a new shoulder located at 1438 cm^−1^. This corresponds to the deformation vibration of the CH_2_ group, followed by the decrease in intensity of the band at 1258 cm^−1^, signaling the epoxy ring opening in NP-N3.

### 3.2. Pickering Emulsion Formation and Polymerization

**Figure 2:**
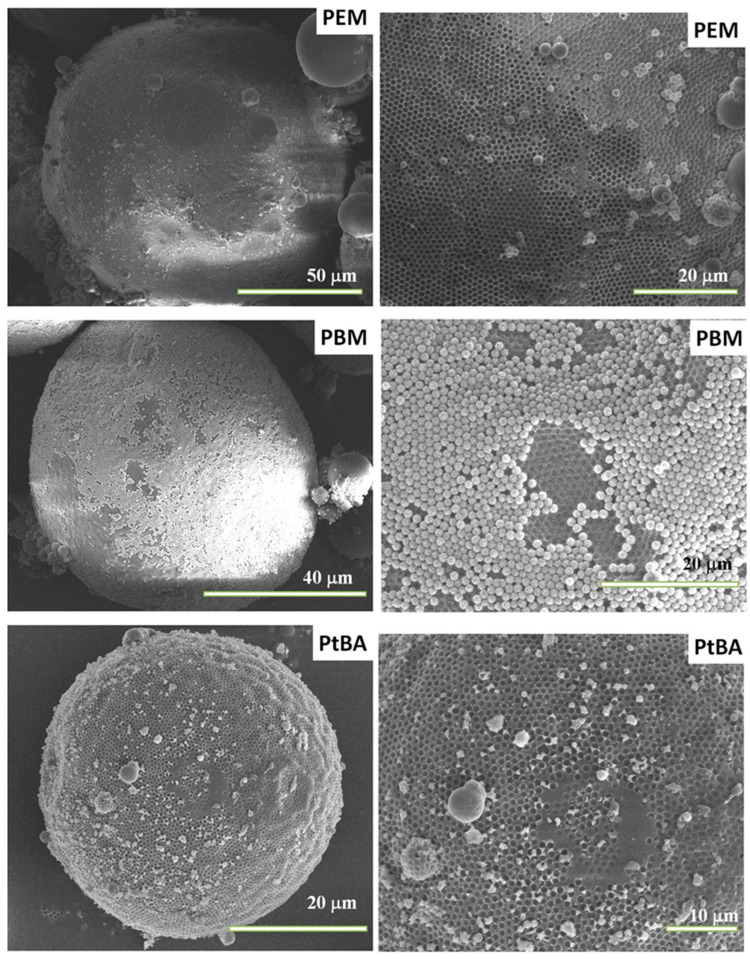
SEM images of the microspheres obtained after the polymerization of the Pickering emulsions of EM, BM and tBA in water, and the corresponding circular traces left by the stabilizing NP-L after Pickering emulsion polymerization.

Pickering emulsions are emulsions stabilized by nanoparticles of different sizes and they were named after S. U. Pickering (1907). Particles stabilize the emulsion by adsorbing irreversibly at the oil/water interface and forming a shield-like monolayer film at the interface [12,13,14]. Some particles adsorb spontaneously at the oil/water interface, but others can be forced to adsorb by kinetic energy input, to overcome the interfacial adsorption barrier [13,15]. Once adsorbed they become trapped at the oil/water interface. It has been experimentally demonstrated that the emulsion droplet sizes and the emulsion phase are determined exclusively by the contact angle *β* of the nanoparticle at the water/oil interface, where the oil can be any organic liquid immiscible with water. In the current case, we convene that the contact angle *β* is the contact angle formed by the nanoparticle with the organic phase, see Figure S2. Our experiment is based on the principles laid forward by Finkle [16], as well as through the experimental observations of Binks and Clint [17] and Aveyard [18], who tried to predict the emulsion phase from the values of the contact angle. We have concluded, also through our experimental investigations [7], that the emulsion phase and oil droplet sizes generated by the spherical nanoparticles depend on the value of the contact angle *β* at the interface. Furthermore, if the oil phase is chosen among the vinyl bearing monomers, the Pickering emulsion can be easily polymerized either thermically or initiated by exposure to UV, such that the nanoparticles trapped at the oil/water interface remain frozen. This enables their observation with scanning electron microscopy (SEM). This is the core principle of the NanoTraPPED method. In this work, we have chosen the following vinyl bearing monomers that are immiscible with water and can be polymerized: MM, EM, BM, DAEMA, tBA and DVB, whose chemical structures are given in Figure S3. For the Pickering emulsion stabilization we have chosen NP-OH of diameters of ≈500 ± 15 nm, shown in the SEM images, which we have subsequently modified, see Figure S1. Upon polymerization PMM, PEM, PBM, PDAEMA and PtBA microspheres covered with nanoparticles are obtained. The PEM, PBM and PtBA microspheres obtained from the Pickering emulsions of the corresponding monomers in water stabilized by the NP-L with the are presented in Figure 2. Those for NP-LCu and NP-LCo are given in Figures S4 and S5. From the inset of the SEM images, in Figure 2, the NPs self-assemble around the Pickering emulsion droplets of the corresponding monomers in water, stabilizing the emulsion, which after the polymerization remain trapped at the interface. Some of the NPs have already fallen, leaving on the surface of the polymer circular traces, whose diameter is direct evidence of their immersion at the interface as it will be discussed next. 

Similarly, the PBM, PDAEMA, PMM, PEM, PtB and PBM microspheres resulting from the Pickering emulsion polymerization of the corresponding monomer emulsions stabilized with NP-Gly and NP-N3 are given in Figures S6 and S7, respectively. Generally, the microspheres obtained in this case are spherical, and their surface is sometimes slightly wavy or wrinkled, which is due to interfacial jamming of nanoparticles.

An overview of the composition of the Pickering emulsions recipes for each monomer as well as the polymerization conditions are given in the Table S2. The surface-trapped nanoparticles can be removed to observe the traces left by the nanoparticles, as we will cover next.

### 3.3. Measuring the Contact Angles—The NanoTraPPED Method

**Figure 3:**
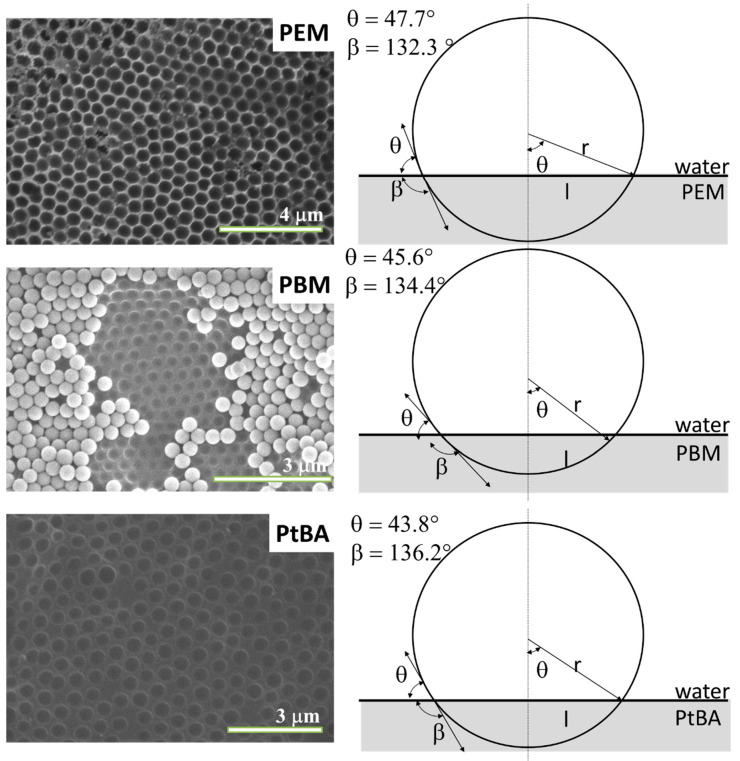
SEM images of the circular traces left by NP-L on the surface of PEM, PBM and PtBA microspheres. The cartoons on the right represent the immersion depth of the nanoparticles at the interface that can be fully described by the contact angle values *θ* with the water and *β* with the organic phase. In the image the *l* represents the diameter of the circular trace determined by SEM, and r represents theradius of the nanoparticle.

The principles of the NanoTraPPED method have been previously discussed [2]. In the current case, the diameter of the circular traces left on the surface of the microparticles obtained via Pickering emulsion polymerization were observed with scanning electron microscopy (SEM) after the removal of the silica nanoparticles by dissolving them with concentrated solution sodium hydroxide, or by simple ultrasonication. The diameter of the circular traces remaining on the surface of the microspheres depend on the immersion depth of the NPs at the interface between water and monomer before polymerization. As previously discussed [2,7], the immersion depth depends on the affinity of the surface of the NPs to the organic phase. The diameter of the circular traces can be geometrically related to the contact angle *β* of the nanoparticle, see Figure S2. $\theta =\sin^{-1}\frac{l}{r}$, *β* = 180° − *θ*, where *l* is the diameter of the circular trace imprinted by the nanoparticle on the surface of the polymer and *r* is the radius of the NPs. The contact angle *β* of the NP-L with PEM, PBM and PtBA, as well as the SEM images from which it was measured, are presented in Figure 3. The results for the NP-LCu and NP-LCo are given in Figures S8 and S9, respectively. It is important to note that the contact angle *β* measured with NanoTraPPED is the contact angle of the NPs at the three-phase line of NP–polymer (NP/P), NP–water (NP/W) and polymer–water (P/W), as indicated by the subscript to the Greek letter γ throughout this work. From Figure 3, it can be qualitatively seen that the *β* of NP-L at the interface increases with the decrease in the polarity of the polymer, where the decrease in polarity in the order PEM > PBM > PtBA is quantitatively ranked according to the ratio of the polarity to the disperse component of the surface energy γPpγPd. The $\gamma_{P}^{p}$ and $\gamma_{P}^{d}$ for each of the polymers used in this work were experimentally determined in a previous work [2].

**Figure 4:**
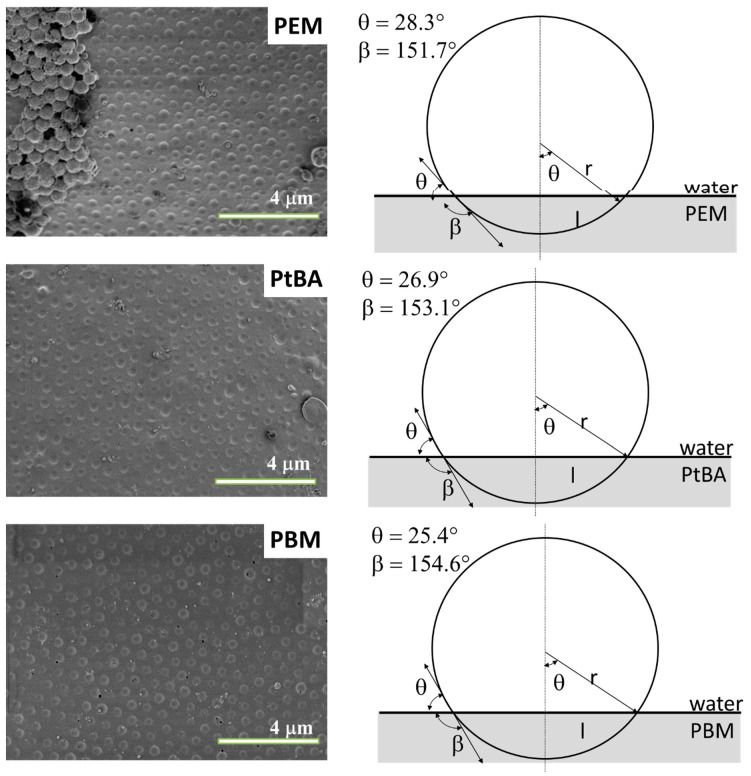
SEM images of the circular traces left by NP-Gly on the surface of PEM, PtBA and PBM microspheres. The cartoons on the right represent the immersion depth of the nanoparticles at the interface that can be fully described by the contact angle values *θ* with the water and *β* with the organic phase. In the image the *l* represents the diameter of the circular trace determined by SEM, and *r* represents the radius of the nanoparticle.

Similarly, the circular traces left on the surface of the polymer microspheres by NP-Gly and NP-N_3_ after surface reactions are given in Figure 4 and Figure S10, respectively. When comparing the contact angle *β* of the same polymers with different nanoparticles, for example NP-L in Figure 3 with NP-Gly in Figure 4, or NP-LCo in Figure S9 with NP-N_3_ in Figure S10, these appear to be significantly different. Whereas, for the same nanoparticle but different polymers the contact angles vary much more subtly; compare the column of images contained within Figure 3, Figure 4, Figures S9 and S10. This proves that the method is extremely sensitive to a change in surface functional groups of NPs, and is less sensitive to the chemistry of the polymer. This can be explained by the fact that the nanoparticle–water, NP/w, interfacial tension unit vector, γ_NP/w_, is larger than that of the nanoparticle–polymer, NP/P, γ_NP/P_. The magnitude of these two interfacial tension unit vectors are the main parameters determining the value of the contact angle of the NPs at the oil/water interface [1]. Therefore, when a surface modification is performed on the NPs, it results in a much stronger response in the contact angle, due to a stronger change in γ_NP/w_ than a change in γ_NP/P_. This is valid for the o/w emulsions, for which NPs are more immersed in water phase than in the organic phase [7]. The opposite should be true for the inverse water-in-oil (w/o) Pickering emulsions. 

The experimental data of the circular hole diameters on the polymer colloidosomes left by the NPs and the calculated contact angles, are summarized in Table S3.

## 4. Discussion

### 4.1. Calculating the Surface Energy and Its Polar and Disperse Components

The surface energy of a surface and its polar and disperse components can be calculated if the contact angles with at least two liquids with known values for surface energy and the components are known. In the current case, the values of *β*—the contact angle of the NP at the interface with the polymer phases are used instead. Furthermore, we have used the Owens–Wendt–Rabel–Kaelble (OWRK) [8,19] model to determine the surface energy components from the contact angle β measured for the NPs with different polymers of known surface energy and components $\gamma_{P/W}^{d}$, $\gamma_{P/W}^{p}$ [2]:

(1) $$\frac{\gamma_{P/W}\left(1+\cos\theta \right)}{2\sqrt{\gamma_{P/W}^{d}}}=\sqrt{\gamma_{NP/W}^{p}}\sqrt{\frac{\gamma_{P/W}^{p}}{\gamma_{P/W}^{d}}}+\sqrt{\gamma_{NP/W}^{d}}$$

**Figure 5:**
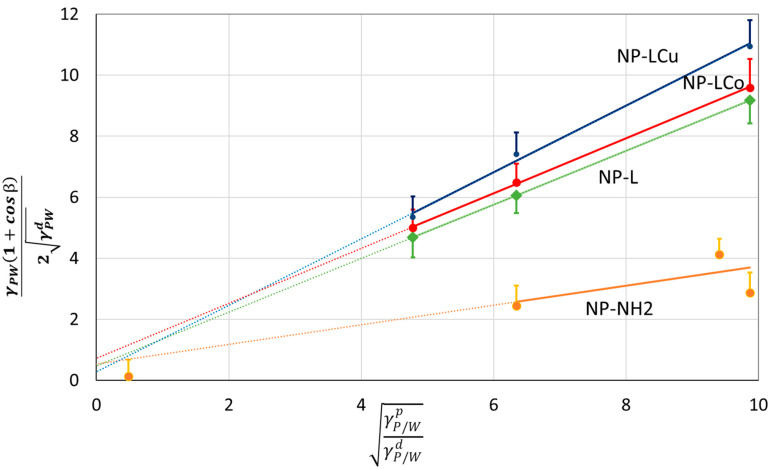
The linear OWRK equation was constructed from the experimental data of the contact angle measured from the circular traces left by the nanoparticles NP-NH_2_, NP-L, NP-LCu and NP-LCo. The error bars represent the standard error and are represented only on the positive or negative side of the measured average value.

**Figure 6:**
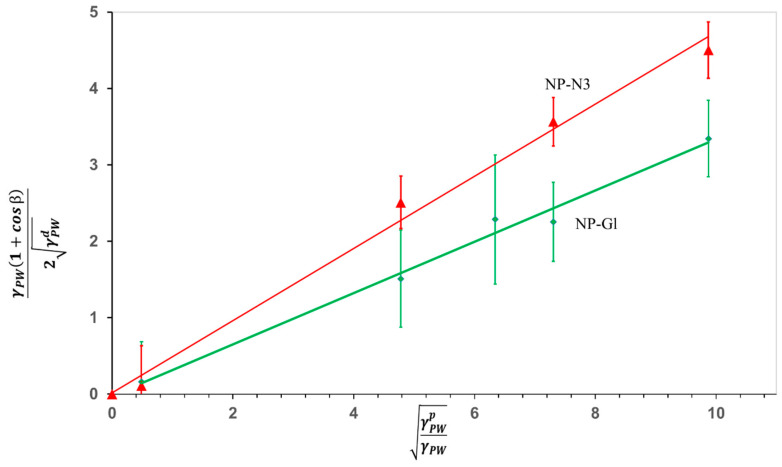
The linear OWRK equation was constructed from the experimental data of the contact angle measured from the circular traces left by the nanoparticles NP-Gly and NP-N_3_. The error bars represent the standard error and are represented both on the positive and negative side of the measured average value.

Thus, by measuring the contact angle β of each nanoparticle with at least three different polymer/water interfaces, we calculated the total surface energy and its components $\gamma_{NP/W}^{p}$, $\gamma_{NP/W}^{d}$ in water by fitting the data to a linear equation where the $\sqrt{\gamma_{NP/W}^{p}}$ is the slope and the $\sqrt{\gamma_{NP/W}^{d}}$ is the intercept, as shown in the Figure 5 for Reaction 1 and Figure 6 for Reaction 2. Interestingly, for Reaction 1, from the data in Figure 5, one can see that the error bars for the NP-L and NP-LCo are overlapping throughout the range of the measurement, which suggests that these values are in fact indistinguishable from one another. On the other hand, the NP-LCu values differ for the latter portion of the curve, with clearly non-overlapping errors. Thus, we conclude that the NP-L and NP-LCo are indistinguishable, while the NP-LCu and NP-NH_2_ could be clearly distinguished from the other groups of data, meaning that they are uniquely determined.

For Reaction 2, the slope of the NP-N_3_ is markedly different from that of NP-Gly, suggesting that the polarity of the former is greater than that of the latter. For the latter portion of the curves in Figure 5 the error bars of the two curves do not overlap, suggesting that the group of data belonging to NP-N_3_ is clearly distinguishable from that of NP-Gly.

### 4.2. Monitoring the Change in the Surface Energy of NPs with NanoTraPPED

The surface energies were determined for each of the NPs after each step of surface functionalization, namely the $\gamma_{NP/W}^{d}$*,* $\gamma_{NP/W}^{p}$ and the values are summarized in Table 1.

From the data presented in the Table 1, for Reaction 1, we can state that the total interfacial energy of the NPs with water, γ_NP/water_, increases in the order NP-NH_2_ < NP-L < NP-LCu < NP-LCo. A higher value of the γ_NP/water_ indicates a decreasing affinity between the NPs and water. Note that it is generally accepted that a vanishingly small interfacial tension indicates total wettability and adhesion of a liquid on a solid surface, or in the case of a liquid biphasic system, a vanishingly small interfacial tension between the liquids indicates a high level of miscibility to total miscibility. Similarly, here, the NP-NH_2_ have the lowest total interfacial energy with water, signaling a good dispersibility of these in water. Although the others are comparable, we predict that the sedimentation of the nanoparticle colloid will be faster (these systems have only kinetic colloid stability) for the NP-LCu and NP-LCo than that of NP-NH_2_ due to the observed difference in the interfacial energy. Here we note that the NanoTraPPED method directly yields the interfacial energies of the NPs, because the contact angle is measured directly at the three-phase line, or at the polymer/water interface. Conversely, for Reaction 2, the γ_NP/water_ decreases with the substitution reaction, where NP-Gly > NP-N_3_. This suggests that the NP-N_3_ are better wetted by water.

Furthermore, the interfacial energies $\gamma_{NP/W}^{d}$, $\gamma_{NP/W}^{p}$ can easily be converted using the combining rules equation [1] into the surface energy of nanoparticles in air $\gamma_{NP}^{d}$, $\gamma_{NP}^{p}:$

(2) $$\gamma_{NP}^{p}={\left(\sqrt{\gamma_{NP/W}^{p}}+\sqrt{\gamma_{W}^{p}}\right)}^{2}$$

Based on the above formula, we have recalculated the values of the surface energies and its disperse and polar components, $\gamma_{NP}^{d}$, $\gamma_{NP}^{p}$ that are given in Table 2.

From the values presented in Table 2, a general trend can be observed, for the Reaction 1, namely the surface energy of the NPs, γ_NP_, increases in the order NP-OH < NP-NH_2_ < NP-L ≤ NP-LCu < NP-LCo. The steepest increase in the surface energy is first observed in Table 2, from NP-OH upon the attachment of 3-(triethoxysilyl)propylamine with the formation of NP-NH_2_ functionality. This increase in the polar component $\gamma_{NP}^{p}$ to the almost doubling of its value, although the -NH_2_ has a smaller dipole moment than the -OH, see Table S4, can be explained by the introduction of a greater number of these functional groups on the surface of the NPs than that of hydroxyl groups. The second greatest change in surface energy can be observed for NP-L after the surface attachment of the ligand by the reaction between the 2,4-dyhydroxy benzaldehyde and the surface amine groups, see Table 2. Again, the change in the polar surface energy component is the steepest, $\gamma_{NP}^{p}$, and is much larger for NP-L than for NP-NH_2_, while the disperse component varies only slightly. This can be explained by the increasing number of polar functional groups; for every ligand attached on the -NH_2_, two more polar -OH moieties (Table S4) are introduced, see Scheme 1A. 

Upon the exposure of the NP-L to a solution of 10^−2^ M of Cu^2+^ and Co^2+^ ions, the metal ion can be either attached to the surface of the NP-L by chemical reaction, coordination and formation of a chemical complex with the Schiff base, or physical adsorption on the negatively charged NP-L surfaces, see the zeta potential value in Table S1. While the surface attachment of cations via physical adsorption is inevitable, the coordinative binding of the metal ions by the ligand L was also demonstrated from the FTIR spectra. The change in the surface energy NP-L with the physisorption and chemical coordination of the of Co^2+^ with the formation of the NP-LCo complex is minimal, and is indistinguishable within the error limit of the method. Only in the case of Cu^2+^ does the formation of the NP-LCu lead to some more notable change in the total surface energy, mainly due to the increase in the polar component, see Table 2. The increase in the polar component with the coordination of the Cu(II) ions could be interpreted by the formation of highly polar coordinative L- > Cu(II) bonds, but this comes at the expense of the elimination of polar functionalities of imine -CH=N and hydroxyls -OH. However, the overall polarity of these interfacial complexes will also depend on their geometry. The only slight change in the polar $\gamma_{NP}^{p}$ component with Co(II) and Cu(II) detected suggests that the transformation of the interfacial ligand and the formation of the interfacial complex does not come with a dramatic surface transformation, but it is still detectable with NanoTraPPED. 

For Reaction 2, see Table 2, the functionalization from NP-OH to NP-Gly produces a stark change in the disperse component of the surface energy, while the functional group replacement from NP-Gly to NP-N_3_ produces only a slight increase in both the polar and disperse energy component such that the total surface energy increases from 77.74 mN/m in the former to 80.9 mN/m in the latter case. Although these observed changes are small, the non-overlapping errors in Figure 6 suggests that these are significant and above the error limit of the method. We further note that in Reaction 2, similarly to Reaction 1, there are subtle changes in the functional groups, as the coordination of metal ions and substitution of glycidyl with azide produces only small changes in the total surface energy or its components. The polar surface energy $\gamma_{NP}^{p}$ component increases in the order NP-OH > NP-Gly > NP-N_3_, which seems to follow the increase in the overall dipole moment of the functional groups, see Table S4. The disperse component due to London dispersive interactions also increases in the same order. 

Another interesting aspect to note is that the overall surface energy of the NPs seems to increase with the increase in the complexity of the molecular structure attached to the surface; that is, any surface modification with an increase in the number of bonds, branching or increase in number of atoms leads to an increase in the total surface energy. 

## 5. Conclusions

In this work, we have demonstrated that the NanoTraPPED method can be deployed in monitoring the chemical transformation taking place at the surface of NPs. For this purposed we have designed two surface reactions that change the surface chemistry either by attachment or coupling of a molecular fragment to the surface or by replacement of a surface functionality. Stark changes in the surface energy of NPs are noted when a large molecular fragment is attached to the surface, such as the attachment of 3-(triethoxysilyl)propylamine, or 2,4-dyhydroxy benzaldehyde, and only minor changes in the surface energies are noted when slight changes in the nature of functional groups are performed, such as the substitution of the similar epoxy moiety with azide, or coordination of metal ions. Nonetheless, the NanoTraPPED proves to be a viable method to monitor the surface reactions and to fully characterize the physicochemical state of NPs.

## Figures and Tables

**Table 1:** Interfacial energies $\gamma_{NP/W}^{d}$, $\gamma_{NP/W}^{p}$ of the NPs adsorbed at the interface calculated with the OWRK equation graphically represented in Figure 5 and Figure 6.

Nanoparticle	γ^p^_NP/water_ (mN/m)	γ^d^_NP/water_ (mN/m)	γ_NP/water_ (mN/m)
* NP-OH	1.05	0.01	1.06
** NP-Gly	0.66	0.1	0.76
NP-N3	0.21	0.02	0.23
* NP-NH_2_	0.13	0.00	0.13
NP-L	0.78	0.22	1.00
NP-LCu	1.16	0.08	1.24
NP-LCo	0.81	0.52	1.33

* values taken from reference [2]. ** values taken from reference [7].

**Table 2:** Surface energies $\gamma_{NP}^{d}$, $\gamma_{NP}^{p}$ of the NPs calculated with the Equation (2).

Nanoparticle	*γ^p^_NP_* (mN/m)	*γ^d^_NP_* (mN/m)	γ _NP_ (mN/m)
* NP-OH	25.40	33.92	59.32
** NP-Gly	26.02	51.72	77.74
NP-N3	27.43	53.47	80.90
*NP-NH_2_	41.96	25.40	67.36
NP-L	59.86	30.83	90.69
NP-LCu	62.96	28.67	91.63
NP-LCo	60.18	33.62	93.80

* values taken from reference [2]. ** values taken from reference [7].

## Data Availability

The data generated in this study are publicly available in an open access repository Open Science Framework (OSF) repository at https://doi.org/10.17605/OSF.IO/TYM28.

## Supplementary Materials

The following are available online at https://www.mdpi.com/article/10.3390/nano13071246/s1, Scheme S1: Surface functionalization reaction scheme, Figure S1: SEM images of the silica NPs before and after functionalization, Figure S2: Cartoon of nanoparticle adsorbed at the oil/water interface, chemical structure of the vinyl bearing monomers, Figure S3: Recipes for Pickering emulsion preparation and polymerization, Figures S4–S7: Microspheres of polymers with the circular traces obtained on their surface after the polymerization of the Pickering emulsion stabilized by different nanoparticles, Figures S8–S10: Diameters of the circular traces on the surface of the microspheres and the contact angles at the interface, Table S1: NP zeta potentials, Table S2: SEM images of microparticles, Table S3: Summary of all hole diameters and contact angles with the polymer, Table S4: calculated values of the electric dipole moments of the functional groups.
