# Supplementary material for: Monitoring the Surface Energy Change of Nanoparticles in Functionalization Reactions with the NanoTraPPED Method
Source: Nanomaterials (Basel). 2023 Mar 31;13(7):1246. doi: 10.3390/nano13071246 (PMC10097230; doi:10.3390/nano13071246)
Supplement: Supplementary file 1 [file nanomaterials-13-01246-s001.zip › nanomaterials-2317464-supplementary.pdf]

nanomaterials

MDPI

Supplementary Materials

Monitoring the Surface Energy Change of Nanoparticles in Functionalization Reactions with the NanoTraPPED Method

Andrei Honciuc * and Oana-Iuliana Negru

Petru Poni Institute of Macromolecular Chemistry, Aleea Gr. Ghica Voda 41A, Iasi 700487, Romania
* Correspondence: honciuc.andrei@icmpp.ro

1. Nanoparticle preparation, functionalization, and characterization

Table S1. Zeta ζ-potential values of the unmodified and modified the silica nanoparticles.

Functional group at the surface	ζ-potential [mV]
NP-OH (starting material NPs)	−53.7 ± 0.5
NP-NH_2	−19.4 ± 0.8
NP-L	−28.6 ± 0.6
NP-L_Cu	−39.6 ± 0.3
NP-L_Co	−40.5 ± 0.2
NPSiCu	−58.4 ± 0.3
NPSiCo	−50.3 ± 0.4

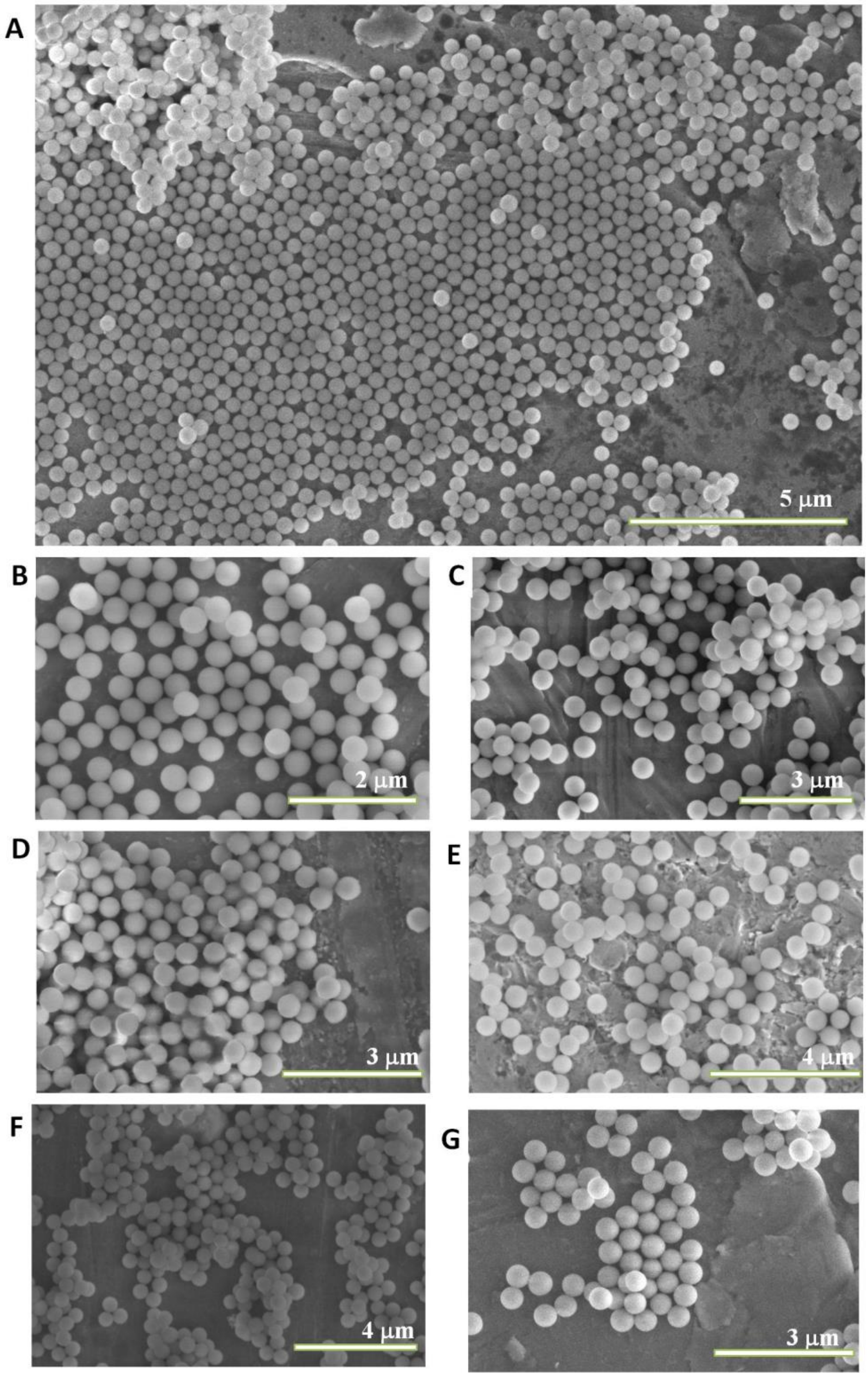

Figure S1. SEM images of the NPs (**A**) NP-OH, (**B**) NP-NH_2, (**C**) NP-Gly, (**D**) NP-N_3, (**E**) NP-L, (**F**) NP-LCu, (**G**) NP-LCo.

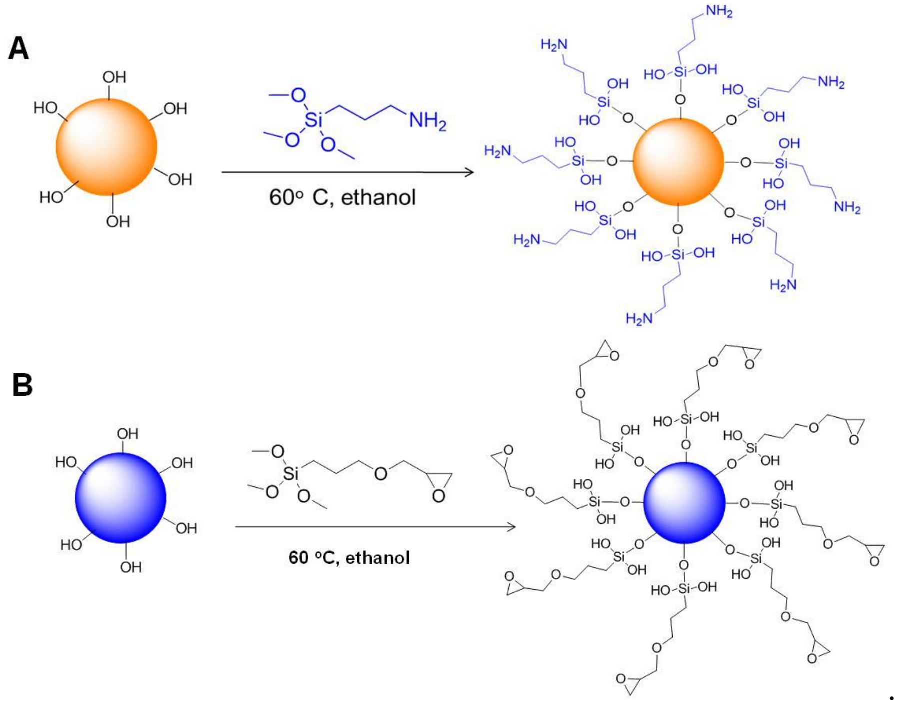

Scheme S1. (A) Synthesis of the NPs has amine surface functionalities NP-NH_2 from the starting NP-OH; **(B)** Synthesis of the NPs has surface functionalities NP-NGly from the starting NP-OH.

2. Pickering emulsion preparation and polymerization

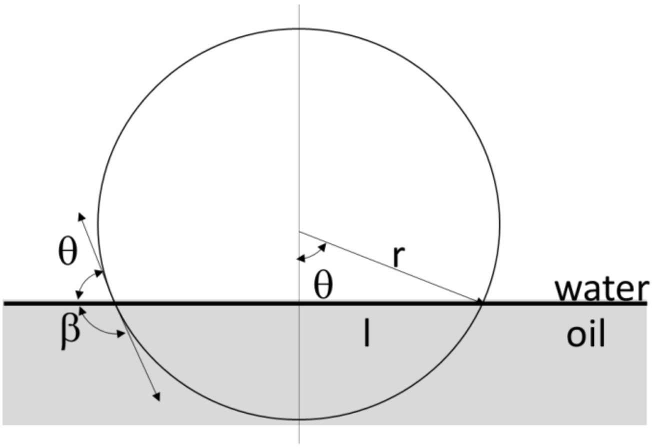

Figure S2. Contact angle is the contact angle formed by the nanoparticle with the organic phase, at the oil/water interface.

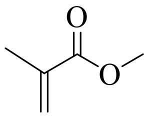
MM

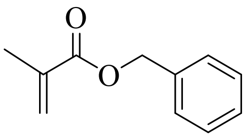
BM

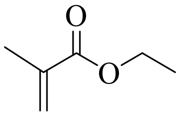
EM

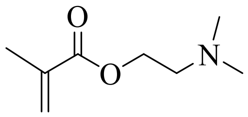
DAEMA

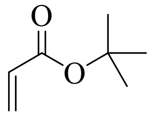
t-BA

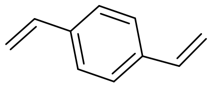
DVB

Figure S3. Vinyl bearing monomers used for the Pickering emulsion formation and polymerization.

Table S2. A summary of the composition of the Pickering emulsions and synthesis of microspheres used in determining the surface energy with the NanoTraPPED method. The acronyms corresponding the monomers were given in the Materials section in the main text.

**Functional Group**	**Monomer 1 mL**	**DVB [mL]**	**BME [mg]**	**H_2O [mL]**	**NP [mg/mL]**	**Sonication [s]**	**Amplitude [%]**
**NP-L**	EM	0.1	20	12	5	15	30
	BM	0.1	20	12	5	15	30
	tBA	0.1	20	12	5	15	30
NP-LCu	EM	0.1	20	12	5	15	30
	BM	0.1	20	12	5	15	30
	tBA	0.1	20	12	5	15	30
NP-LCo	EM	0.1	20	12	5	15	30
	BM	0.1	20	12	5	15	30
	tBA	0.1	20	12	5	15	30
NP-N3	BM	0.1	20	12	5	15	30
	MM	0.1	20	12	5	15	30
	EM	0.1	20	12	5	15	30
	DAEMA	0.1	20	12	5	15	30

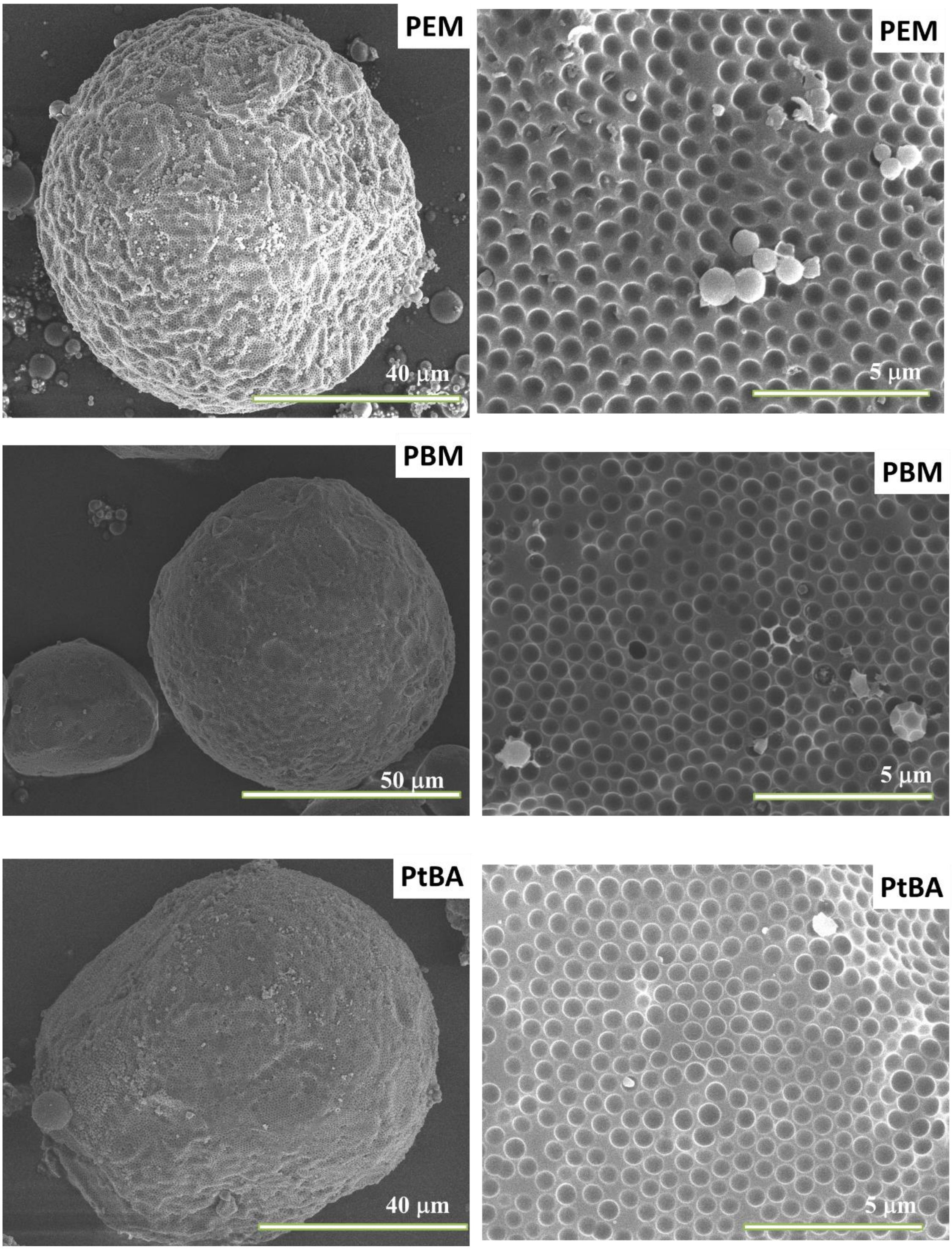

Figure S4. Microspheres of PEM, PBM, and PtBA with the circular traces obtained on their surface after the polymerization of the Pickering emulsion stabilized by NP-LCu.

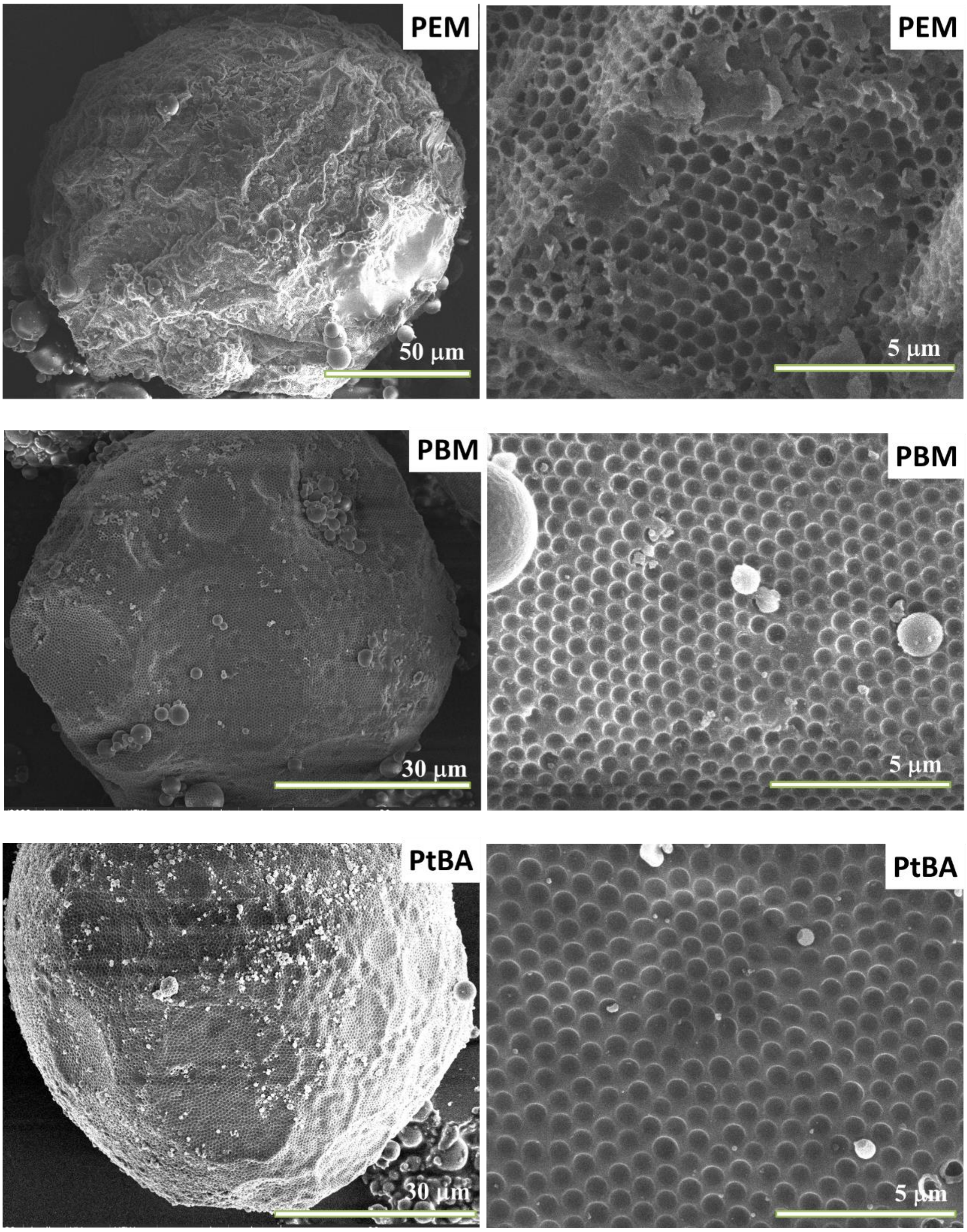

Figure S5. Microspheres of PEM, PBM, and PtBA with the circular traces observed on their surface after polymerization of the Pickering emulsion stabilized by NP-LCo.

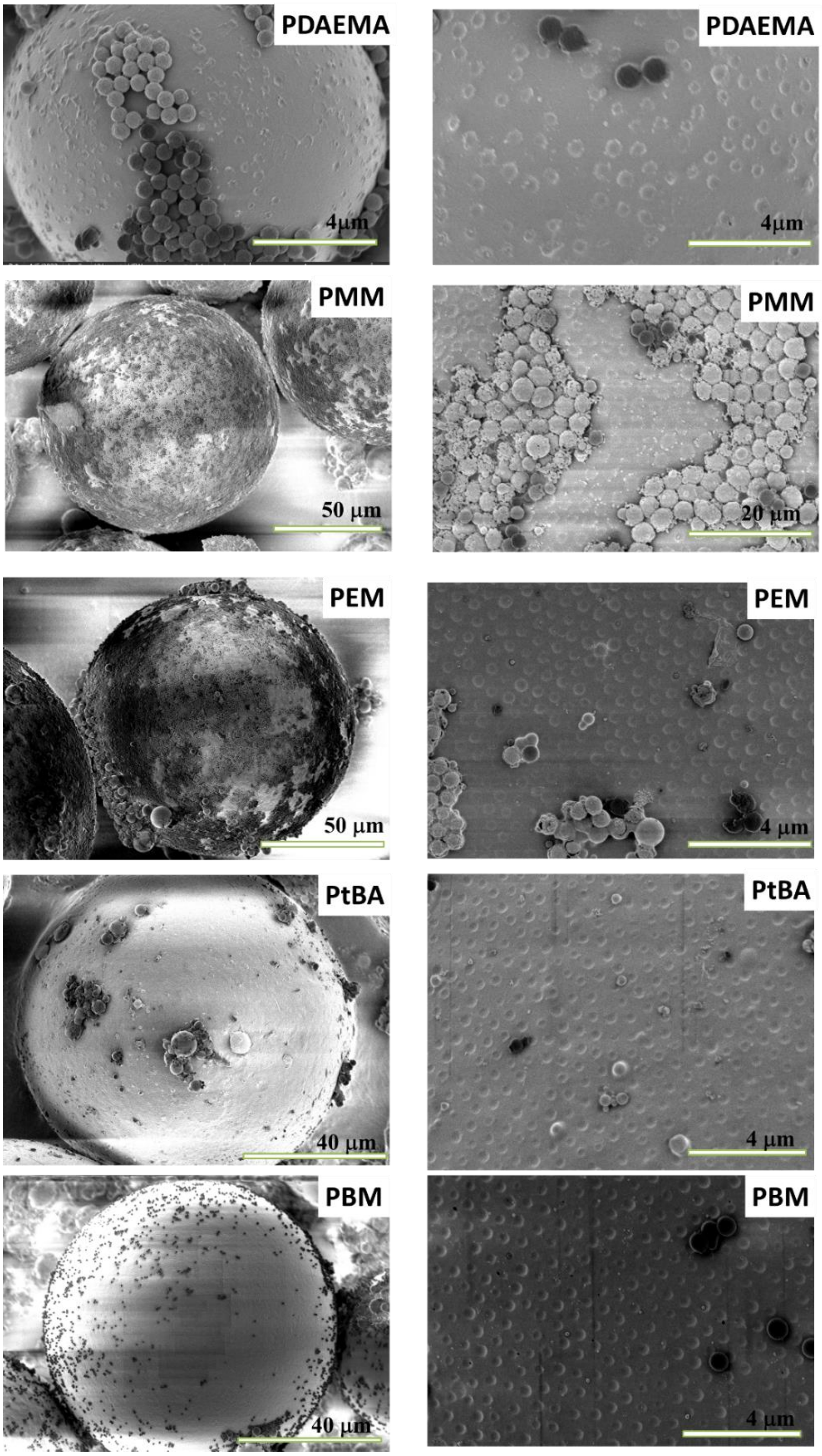

Figure S6. Microspheres of PDAEMA, PMM, PEM, PtBA and PBM with the circular traces observed on their surface after polymerization of the Pickering emulsion stabilized by NP-Gly.

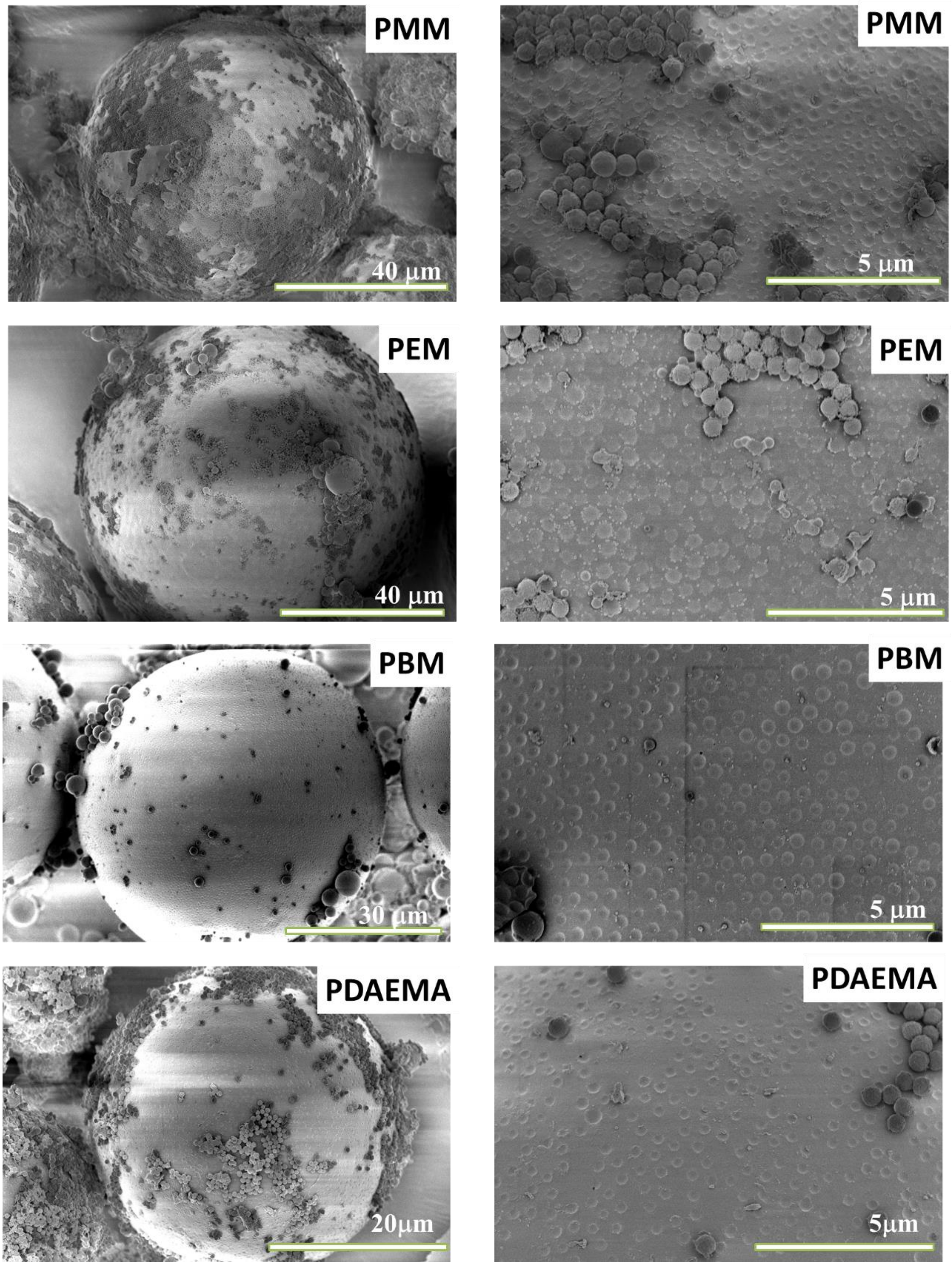

Figure S7. Microspheres of PMM, PEM, PBM and PDAEMA, with the corresponding circular traces observed after the polymerization of the Pickering emulsion stabilized by NP-N_3.

3. NanoTraPPED method

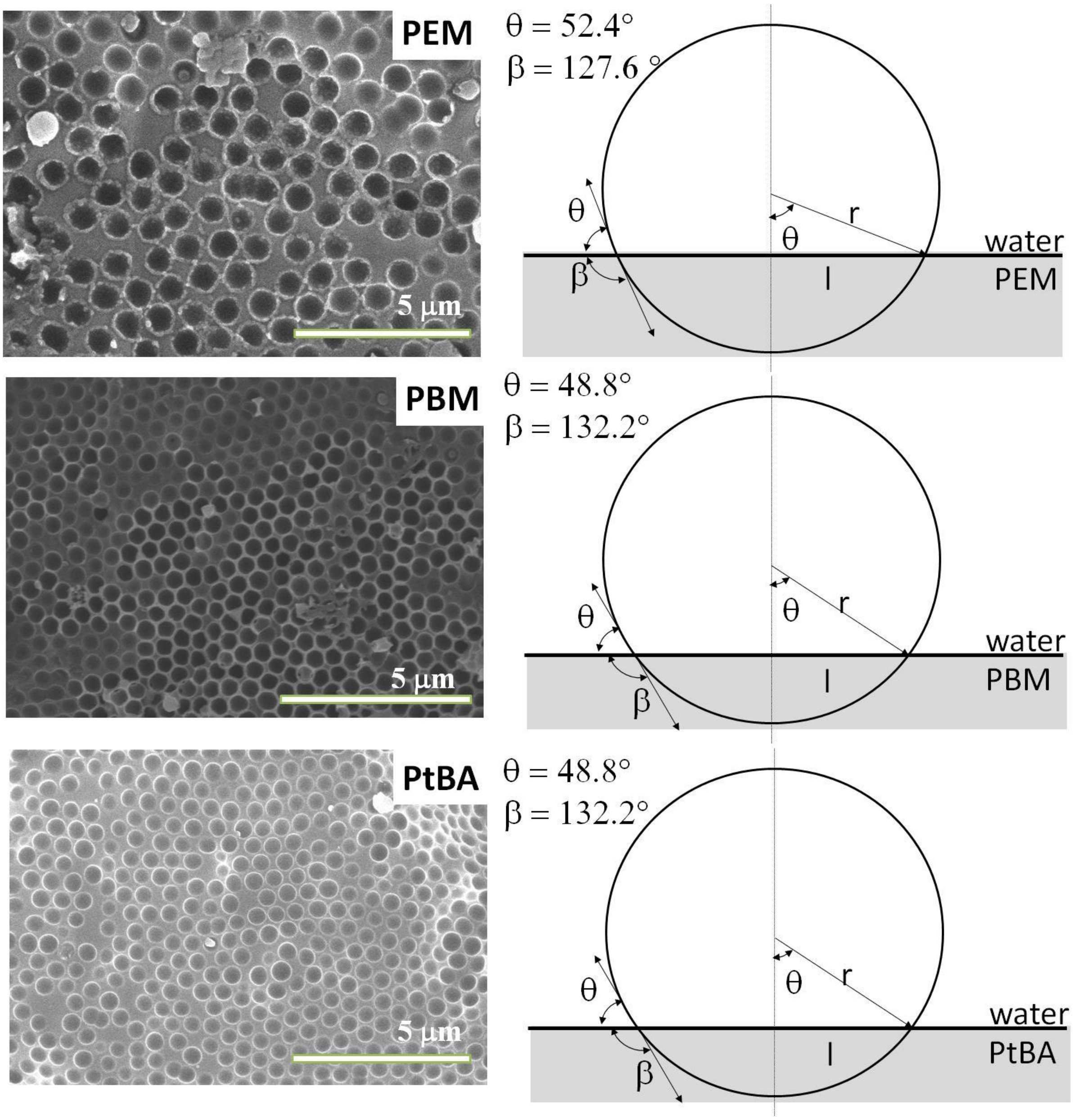

Figure S8. SEM images of the NP-LCu with PEM, PBM, and PtBA microspheres obtained after the Pickering emulsion polymerization and the circular traces left by the stabilizing nanoparticles. In the image the l represents the diameter of the circular trace determined by SEM, r—radius of the nanoparticle, θ—the contact angle of the NP at the interface with the water phase and β—the contact angle of the NP at the interface with the polymer phase.

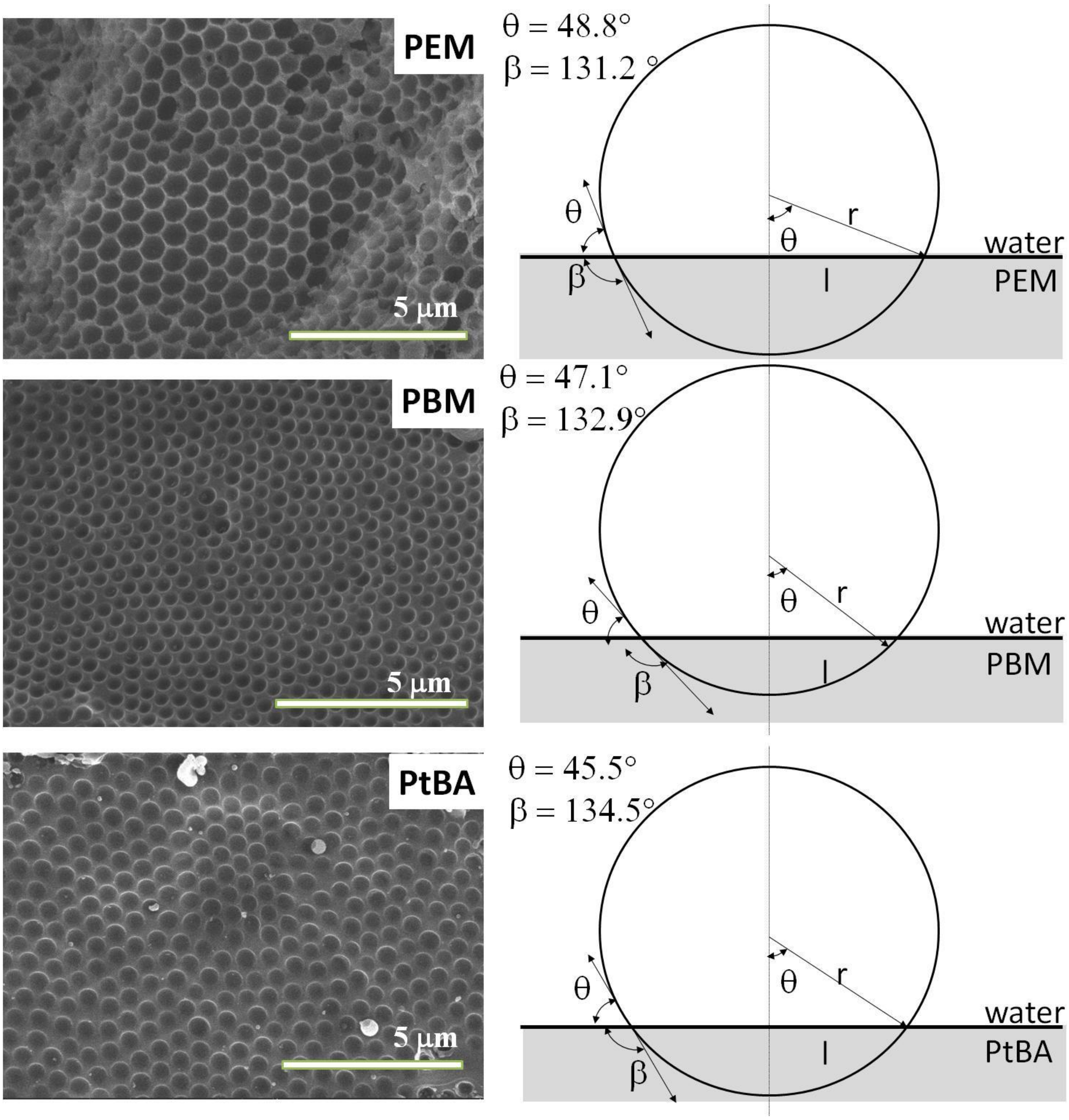

Figure S9. SEM images of the NP-LCo with PEM, PBM, and PtBA microspheres obtained after the Pickering emulsion polymerization and the circular traces left by the stabilizing nanoparticles. In the image the l represents the diameter of the circular trace determined by SEM, r—radius of the nanoparticle, θ—the contact angle of the NP at the interface with the water phase and β—the contact angle of the NP at the interface with the polymer phase.

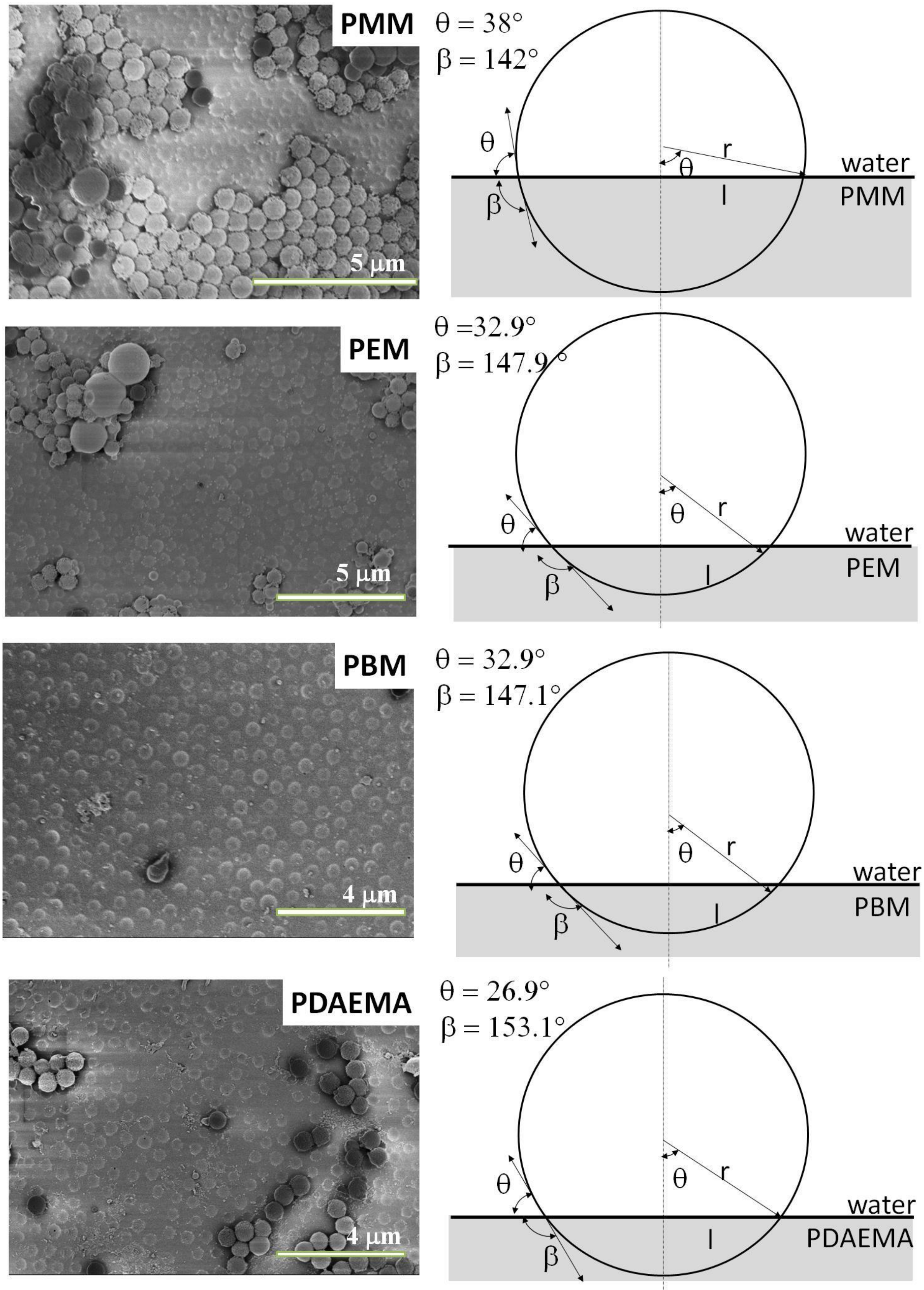

Figure S10. SEM images of the NP-N3with PMM, PEM, PBM, and PDAEMA microspheres obtained after the Pickering emulsion polymerization and the circular traces left by the stabilizing nanoparticles. In the image the l represents the diameter of the circular trace determined by SEM, r—radius of the nanoparticle, θ—the contact angle of the NP at the interface with the water phase and β—the contact angle of the NP at the interface with the polymer phase.

Table S3. Summary of all hole diameter, contact angle with the polymer.

Functional Group	Polymer	Hole diameter [nm]	β °
NP-L	PBM	330 ± 2	134.42 ± 0.64
	PtBA	320 ± 2	136.16 ± 0.55
	PEM	342 ± 2	132.60 ± 0.71
NP-LCu	PBM	380 ± 2	131.45 ± 0.55
	PtBA	380 ± 2	131.45 ± 0.58
	PEM	400 ± 2	127.91 ±0.78
NP-LCo	PBM	370 ± 1	132.27 ± 0.46
	PtBA	360 ± 2	133.95 ± 0.55
	PEM	380 ± 3	130.54 ± 1.07
NP-N_3	PMM	340 ± 3	142.00 ± 0.72
	PDAEMA	250 ± 2	153.08 ± 0.36
	PBM	300 ± 2	147.09 ± 0.39
	PEM	300 ± 2	147.09 ± 0.45

Table S4. Ranking of the polarity of functional groups by their dipole moment according to the calculated values of the electric dipole moments from the Computational Chemistry Comparison and Benchmark DataBase of the National Institute of Standards and Technology (https://cccbdb.nist.gov/dipole2.asp). The calculations were performed with density functional theory DFT, with density functionalsPBE1PBE and 6-31 + G** basis set.

Functional group	Calculated dipole moment (Debye)
CH_3-OH	1.888
CH_3-NH_2	1.463
Epoxy	2.081
CH3-N3	2.459
